# Access to medicines and diagnostic tests integral in the management of diabetes mellitus and cardiovascular diseases in Uganda: insights from the ACCODAD study

**DOI:** 10.1186/s12939-017-0651-6

**Published:** 2017-08-24

**Authors:** Davis Kibirige, David Atuhe, Leaticia Kampiire, Daniel Ssekikubo Kiggundu, Pamela Donggo, Juliet Nabbaale, Raymond Mbayo Mwebaze, Robert Kalyesubula, William Lumu

**Affiliations:** 1 Department of Medicine, Uganda Martyrs Hospital Lubaga, P.O.BOX 7146 Kampala, Uganda; 2 Department of Medicine, Case Hospital Kampala, Kampala, Uganda; 3 Infectious Disease Research Collaboration (IDRC), Kampala, Uganda; 4 Nephrology unit, Mulago National Referral and Teaching Hospital, Kampala, Uganda; 5 Department of Medicine, Lira Regional Referral Hospital, Lira, Uganda; 6 Division of Adult Cardiology, Uganda Heart Institute, Kampala, Uganda; 7 Department of Medicine, St. Francis hospital Nsambya, Kampala, Uganda; 8 Departments of Physiology and Medicine, Makerere University College of Health Sciences, Kampala, Uganda; 9 Department of Medicine, Mengo Hospital, Kampala, Uganda

**Keywords:** Availability, Cost, Affordability, Diabetes mellitus, Cardiovascular diseases, Low and middle income countries

## Abstract

**Background:**

Despite the burgeoning burden of diabetes mellitus (DM) and cardiovascular diseases (CVD) in low and middle income countries (LMIC), access to affordable essential medicines and diagnostic tests for DM and CVD still remain a challenge in clinical practice. The Access to Cardiovascular diseases, Chronic Obstructive pulmonary disease, Diabetes mellitus and Asthma Drugs and diagnostics (ACCODAD) study aimed at providing contemporary information about the availability, cost and affordability of medicines and diagnostic tests integral in the management of DM and CVD in Uganda.

**Methods:**

The study assessed the availability, cost and affordability of 37 medicines and 19 diagnostic tests in 22 public hospitals, 23 private hospitals and 100 private pharmacies in Uganda. Availability expressed as a percentage, median cost of the available lowest priced generic medicine and the diagnostic tests and affordability in terms of the number of days’ wages it would cost the least paid public servant to pay for one month of treatment and the diagnostic tests were calculated.

**Results:**

The availability of the medicines and diagnostic tests in all the study sites ranged from 20.1% for unfractionated heparin (UFH) to 100% for oral hypoglycaemic agents (OHA) and from 6.8% for microalbuminuria to 100% for urinalysis respectively. The only affordable tests were blood glucose, urinalysis and serum ketone, urea, creatinine and uric acid. Parenteral benzathine penicillin, oral furosemide, glibenclamide, bendrofluazide, atenolol, cardiac aspirin, digoxin, metformin, captopril and nifedipine were the only affordable drugs.

**Conclusion:**

This study demonstrates that the majority of medicines and diagnostic tests essential in the management of DM and CVD are generally unavailable and unaffordable in Uganda. National strategies promoting improved access to affordable medicines and diagnostic tests and primary prevention measures of DM and CVD should be prioritised in Uganda.

## Background

Globally, the prevalence of diabetes mellitus (DM) and cardiovascular diseases (CVD) has significantly reached epidemic levels especially in the low and middle income countries (LMIC) [1, 2]. This poses a colossal public health threat. Both DM and CVD adversely affect productivity, reduce quality of life, increase rates of mortality and cause a massive economic strain to a nation’s health systems, families and individuals [3].

Challenges like suboptimal screening, diagnosis and management of DM and CVD coupled with low access to affordable essential medicines and diagnostic tests remain frequent in clinical practice in LMIC [4–11]. Improved access to affordable essential medicines and diagnostic tests is an integral component of optimal management of DM and CVD [12]. This directly reduces morbidity and mortality due to DM and CVD.

Due to significant socio-economic and lifestyle changes coupled with the drastic population growth, Uganda is currently experiencing an epidemiological transition from communicable diseases (CD) like tuberculosis and HIV to non communicable diseases (NCD) like DM and hypertension (HT) [13]. Two recently concluded nationwide representative studies to determine the burden of DM [14] and HT [15] in Uganda using the WHO STEP-wise methodology documented the prevalence of DM and HT of 1.4 and 26.4% respectively, with the majority of the participants unaware of their condition. Heart diseases notably hypertensive heart disease, rheumatic heart disease and dilated cardiomyopathy and related complications like acute heart failure and atrial fibrillation are frequently encountered in clinical practice in Uganda [16–18].

Despite the increasing burden of DM and CVD in Uganda, the structuring of the health system impedes provision of optimal DM and CVD care. The majority of lower tier public hospitals (health centres 1, 2, 3 and 4) which are easily accessible to the general population are more oriented towards management of CD as opposed to NCD. Patients with NCD are often referred to higher tier public hospitals (district referral or national referral hospitals) and the costly private hospitals for further management. Preliminary findings from 1 multicentre study reported that the majority of these lower tier Ugandan public hospitals lacked the recommended national and international guidelines of management of NCDs and essential medicines and diagnostic tests [19].

There is limited contemporary data about the availability, cost and affordability of medicines and diagnostic tests integral in the management of DM and CVD in Uganda. Annually, the Medicines Transparency Alliance conducts an assessment of availability and affordability of 40 essential medicines for CD and NCD in the 4 regions of Uganda using the World Health Organization (WHO) and Health Action International (HAI) standardised method. The most recent survey done in May to June 2015 assessed only 5 NCD drugs (Glibenclamide 5 mg, Metformin 500 mg, Nifedipine retard 20 mg, Furosemide 40 mg and Propranolol 40 mg) in 112 public, private and private not-for-profit health facilities. The availability of glibenclamide ranged from 36% in rural private hospitals to 80% in urban private health facilitates while the availability of metformin ranged from 36% in rural private for profit health facilities to 90% in urban public health facilities. Low availability of nifedipine and Propranolol was noted in all the public hospitals (≤55%) [20].

Using the WHO and HAI standardised method for surveying medicine prices in LMIC [21], the ACCODAD study sought to add to the existing information about extent of availability, cost and affordability of medicines and diagnostic tests of NCD in Uganda and in LMIC. This information will be pivotal in influencing the formulation and implementation of national policies aimed at improving access to affordable medicines and diagnostic tests for DM and CVD.

## Methods

### Study settings and selection of study sites

The ACCODAD study was conducted from 15th January 2017 to 28th February 2017 in 22 public hospitals, 23 private hospitals and 100 privately owned pharmacies. The health units were selected from each of the 4 regions of Uganda (central, western, eastern and northern) using random sampling method from the hospital and private pharmacy registries of the Ministry of Health and National Drug Authority (NDA), Republic of Uganda respectively.

The total number of public and private hospitals in Uganda is 155. Two of these are national referral hospitals, 14 are regional referral hospitals and 139 are general hospitals. In terms of ownership, 65 are government owned, 63 private not for profit (PNFP) and 27 are private for profit [22]. The public hospitals offer free medical care to all patients. They procure all their drugs, laboratory tests and medical equipment from one central national procurement institution called the National Medical Stores (NMS). The NMS purchases the essential drugs and diagnostic tests from qualified private suppliers through a locally publicised tender process. Nevertheless, recurrent drug stock outs and unavailability of key diagnostic tests remains a key challenge in the public hospitals. This compels patients to seek medical treatment from the costly privately owned hospitals, clinics and pharmacies. These procure their medicines and diagnostic tests from several private distributors. The NDA registry has a total of 599 registered privately owned retail pharmacies and 90 private hospital pharmacies dealing in human medicines. The majority of the private pharmacies (>70%) are located in the central region [23].

The public hospitals, private hospitals and private pharmacies where the data was collected accounted for 34, 26 and 15% of total national registered public hospitals, private hospitals and private pharmacies respectively. The majority of the study sites were selected from the central region of the country (*N* = 83, 57.2%) because it has the greatest number of registered hospitals and privately owned pharmacies. Study sites selected from the eastern, western and northern region accounted for 15.9, 17.9 and 9% respectively.

### Sample size estimation

Basing on one of the primary objectives of the ACCODAD study i.e. to determine the availability of the medicines and diagnostic tests of interest, the availability of 4 key medicines in DM and CVD management (intermediate insulin or insulitard®, losartan, simvastatin and isosorbide mononitrate) of 10% as reported by the study performed in Western Cameroon was used as the prevalence (P) [6]. Using the formula: n = Z2P (1-P)/d2 where Z (normal value corresponding to the 95% confidence interval) =1.96, *P* = 0.1 and d = 0.05 (desired precision of estimation), a sample size of 138 health units (hospitals and private pharmacies) was obtained. The study sample size was however, increased to 145.

### Data collection

For the ACCODAD study, we collected information about 37 medicines and 19 diagnostic tests significant in the management of DM and CVD as highlighted by the Ugandan local guideline and several international guidelines as highlighted below. The CVD of interest were hypertension, coronary artery disease, stroke, dilated cardiomyopathy, rheumatic heart disease, atrial fibrillation, peripheral arterial disease, venous thromboembolism and related complications like heart failure.

The selected medicines of interest are part of the WHO essential medicines list for treatment of chronic diseases in LMICs [24] and are recommended in the management of DM and CVD by the 2012 Uganda clinical guidelines [25], the 2017 American Diabetes Association guidelines of standard of care of DM and related CVD [22] and the recent European Society of Cardiology (ESC) guidelines of management of atrial fibrillation, acute myocardial infarction, acute heart failure [26–29].

The respective medicine categories and medicines of interest were oral hypoglycaemic agents (OHA) which included metformin 1 g and 500 mg, glibenclamide 5 mg, glimepiride 2 mg and pioglitazone 30 mg, angiotensin II receptor blockers (ARB) which included losartan 50 mg and telmisartan 40 mg, angiotensin converting enzyme inhibitors (ACEI) which included captopril 25 mg, thiazide diuretics (D) which included bendrofluazide 5 mg, ARB-D which included losartan-hydrochlorothiazide 50/12.5 mg and telmisartan-hydrochlorothiazide 40/12.5 mg, loop diuretics which included oral furosemide 40 mg and i.v. furosemide 20 mg, calcium channel blockers (CCB) which included nifedipine 10 mg and 20 mg and amlodipine 5 mg and 10 mg, statins which included simvastatin 20 mg, atovastatin 20 mg and rosuvastatin 10 mg, anti platelet drugs which included cardiac aspirin 75 mg and Clopidogrel 75 mg, low molecular weight heparin which included enoxaparin 60 mg and 80 mg and beta blockers which included atenolol 50 mg, bisoprolol 5 mg, nebivolol 5 mg and carvedilol 6.25 mg. Other surveyed medicines included: parenteral benzathine penicillin, oral digoxin, warfarin, spironolactone, hydralazine, soluble insulin, intermediate insulin, pre mixed insulin, isosorbide mononitrate and unfractionated heparin (UFH).

The selected diagnostic tests are part of the WHO minimum workup tests for assessment and management of cardiovascular (CV) risk [24] and are also recommended by the 2017 American Diabetes Association guidelines of standard of care of DM and related CVD [22] and the recent European Society of Cardiology (ESC) guidelines of management of atrial fibrillation, acute myocardial infarction, acute heart failure [26–29]. These included: lipid profile, glycated haemoglobin (HbA1c), serum uric acid, serum troponin, coagulation profile, thyroid function tests, serum creatinine, serum urea, serum electrolytes, serum ketones, microalbuminuria, complete blood count, serum natriuretic peptides, electrocardiography (ECG), echocardiography (ECHO), chest X ray, liver function tests and urinalysis.

Data was collected using a pre tested questionnaire based on the WHO and HAI standardised methods of assessing medicine prices, availability and affordability in LMIC [21] from 15th January 2017 to 28th February 2017. The data collection team underwent a brief training before commencement of the study to improve quality and standardisation of data.

Information about the availability of diagnostic tests and any medicine in the respective medicine category was obtained. The cost of performing each diagnostic test and the monthly cost of the recommended dose of the available lowest priced generic (LPG) medicine was obtained in Uganda shillings (UgX) and then converted to US dollars (USD) using the existing exchange rate at the time of data collection (1 USD = 3600 UgX). The obtained costs of the medicines were the retail prices charged directly to the patients at the respective pharmacies of the private hospitals and private pharmacies. The cost of the medicines in the public hospitals was not obtained since medical care is offered free of charge.

### Data analysis

Availability of the medicines and diagnostic tests was assessed by calculating the proportion of hospitals and private pharmacies in which any desired dose of the medicine and diagnostic test was present on the day of data collection at the study site. We defined availability as low, moderate or high when the medicines and diagnostic tests of interest were available in <50, 50–79 and ≥80% of the study sites respectively. Availability of the selected medicines and diagnostic tests was compared between the study sites to determine any statistically significant difference which was defined as a *p* value of <0.05. The cost of the available LPG medicine was compared to the cost of the available originator medicine.

The obtained unit retail prices of the medicines in USD were converted to a median price ratio (MPR) by dividing the median local price by an international reference price (IRP). The IRP is obtained from the Management Sciences for Health International Drug Price Indicator Guide which reports median prices of high quality multisource medicines offered to LMIC countries by different suppliers. The MPR is used to express how much greater or less the median local medicine price is than the IRP. An MPR of 3 would mean that the local medicine price is three times greater than the IRP. For patients’ medicine prices, MPR ≤ 1.5 were considered reasonable pricing [30].

Affordability was estimated by calculating the number of days’ wages required to purchase a one month course of treatment or pay for a specific diagnostic test using the average salary of the lowest paid government worker in USD. Medicines and diagnostic tests that cost ≤3 days’ wages were considered affordable. The exchange rate of the local currency (UgX) to USD used was the commercial “buy” rate at the time of data collection of 1 USD = 3600 UgX. The lowest paid government worker at the time of the study (scale U8 lower-non formal education teachers) earned a gross salary of 198,793 UgX (USD 55.2). After tax deductions, this translated to a net salary of 139,155 UgX (USD 38.7) per month or 4638.5 UgX (1.3 USD) daily [31].

## Results

### Availability of the medicines

Low, moderate and high availability was documented in 5 (23.8%), 3 (14.3%) and 13 (61.9%) of the 21 surveyed medicines categories respectively. The availability of the surveyed medicine categories ranged from 20.1% for unfractionated heparin (UFH) to 100% for OHA. High availability was noted for the majority of the key medicine categories in the management of hypertension and cardiac diseases i.e. ARBs, ACEI, D, CCB, statins, anti platelet drugs and beta blockers. None of the insulin types was of high availability. Soluble, intermediate and pre mixed insulin was available in 68.8, 34.7 and 60.1% of all the study sites respectively. Isosorbide nitrate, UFH and LMWH were all of low availability (summarised in Table 1).

**Table 1:** Availability of all the DM and CVD medicines and diagnostic tests in all the study sites

Medicines (*N* = 21 classes)	Availability (%)	Diagnostic tests (*N* = 19)	Availability (%)
UFH	20.1	Microalbuminuria	6.8
Isosorbide mono nitrate	27.8	Serum ketones	11.4
LMWH	31.9	Serum natriuretic peptides	11.4
Intermediate insulin	34.7	Echocardiography	34.1
Hydralazine	47.2	Coagulation profile	36.4
Pre mixed insulin	60.1	Serum troponin testing	43.2
Warfarin	64.6	HbA1c testing	43.2
Soluble insulin	68.8	Thyroid function tests	43.2
ARB-thiazide diuretics	82.6	Electrocardiography	54.6
Digoxin	84.0	Uric acid testing	56.8
Statins	84.0	Lipid profile	65.9
ARB	86.8	Creatinine	86.4
Spironolactone	87.5	Urea	86.4
Benzathine penicillin	87.5	Serum electrolytes	88.6
Furosemide	89.6	Chest X-ray	88.6
Thiazide diuretics	94.4	Liver function tests	95.5
Anti platelet drugs	95.1	Glucometers	97.7
ACEI	96.5	CBC testing	97.7
Beta blockers	97.2	Urinalysis	100
CCB	99.3		
OHA	100		

*UFH* Unfractionated heparin, *LMWH* Low molecular weight heparin, *ARB* Angiotensin II receptor blockers, *ACEI* Angiotensin converting enzyme inhibitors, *CCB* Calcium channel blockers, *OHA* Oral hypoglycaemic agents, *HbA1c* Glycated haemoglobin

### Availability of the diagnostic tests

With regard to the 19 diagnostic tests of interest, 8 (42.1%) were of low availability, 3 (15.8%) were of moderate availability and 8 (42.1%) were of high availability. The availability ranged from 6.8% for microalbuminuria to 100% for urinalysis. Apart from electrocardiography (ECG) and lipid profile testing which were available in only 54.6 and 65.9% of the study hospitals, the rest of the recommended WHO minimum tests for DM and CVD workup were of high availability (random blood glucose tests-97.7%, serum electrolytes-88.6% and urinalysis-100%). Glycated haemoglobin (HbA1c) tests, a key test in DM diagnosis and monitoring of glycaemic control in diabetes care was available in only 43.2% of the study hospitals. The majority of vital tests in cardiac evaluation (echocardiography, coagulation profile, serum natriuretic peptides and troponin tests) were of low availability (summarised in Table 1).

### Comparison of the availability of selected medicines in the different study sites

There were significant differences in the availability of key medicines documented in the public hospitals and the private hospitals and pharmacies. Low availability was noted for these medicine categories in public hospitals compared to the private hospitals and pharmacies: ARBs, ARB-thiazide diuretics, statins, warfarin, LMWH, UFH and nitrates (summarised in Table 2).

**Table 2:** Comparison of the availability of the DM and CVD medicines between the public hospitals, private hospitals and pharmacies

Availability of the medicines in %				
Medicines	Private hospitals (*n* = 23)	Public hospitals (*n* = 22)	Private pharmacy (*n* = 100)	*P* value
A: Medicines with a statistically significant difference between study sites				
ARBs	87.0	31.8	99.0	<0.001
ARB-thiazide diuretics	78.3	27.3	96.0	<0.001
Statins	87.0	18.2	98.0	<0.001
Warfarin	65.2	18.2	74.8	<0.001
Soluble insulin	100	81.8	58.6	<0.001
Thiazide diuretics	82.6	95.5	97.0	0.025
Spironolactone	91.3	68.2	90.9	0.012
LMWH	47.8	9.1	33.3	0.018
UFH	39.1	4.6	19.2	0.014
Isosorbide mono nitrate	30.4	4.6	32.3	0.030
B: Medicines with no statistically significant difference between study sites				
Captopril	91.3	95.5	98.0	0.277
CCB	95.7	100	100	0.071
Beta blockers	95.6	100	97.0	0.650
Anti platelet drugs	91.3	91.0	97.0	0.317
Digoxin	91.3	81.2	82.3	0.579
Pre mixed insulin	69.6	77.3	54.1	0.080
Intermediate insulin	43.5	27.3	34.3	0.516
Benzathine penicillin	91.3	100	83.8	0.097
Loop duiretics	91.3	100	86.9	0.182
Hydralazine	65.2	45.6	43.4	0.166
OHA	100	100	100	1

*UFH* Unfractionated heparin, *LMWH* Low molecular weight heparin, *ARB* Angiotensin II receptor blockers, *ACEI* Angiotensin converting enzyme inhibitors, *CCB* Calcium channel blockers, *OHA* Oral hypoglycaemic agents

### Comparison of the availability of selected diagnostic tests in public and private hospitals

With regard to the diagnostic tests, a statistically significant difference in the availability of lipid profile, HbA1c, uric acid, troponin, coagulation profile and thyroid function tests was noted in the surveyed public hospitals compared to the private hospitals. All the documented tests were of low availability in the public hospitals. Tests for serum ketones, microalbuminuria, serum natriuretic peptides and ECHO were of low availability regardless of the study site (summarised in Table 3).

**Table 3:** Comparison of the availability of the DM and CVD diagnostic tests between the private and public hospitals

Availability of the tests (%)			
Test	Private hospitals (*n* = 23)	Public hospitals (*n* = 24)	*P* value
A: Diagnostic tests with a statistically significant difference between study sites			
Lipid profile	86.4	45.5	0.004
HbA1c	63.6	22.7	0.006
Uric acid	77.3	36.4	0.006
Serum troponin	68.2	18.2	0.001
Coagulation profile	59.1	13.6	0.002
Thyroid function tests	72.7	13.6	<0.001
B: Diagnostic tests with no statistically significant difference between study sites			
Serum creatinine	95.5	77.3	0.079
Serum urea	95.5	77.3	0.079
Glucometers	100	95.5	0.312
Serum electrolytes	95.5	81.8	0.154
Serum ketones	13.6	9.1	0.635
Microalbuminuria	4.6	9.1	0.550
CBC	95.5	100	0.312
Serum natriuretic peptides	18.2	4.6	0.154
Echocardiography	45.5	22.7	0.112
Chest X-ray	95.5	81.8	0.154
Liver function tests	100	90.9	0.148
Electrocardiography	54.5	54.5	1
Urinalysis	100	100	NA

*HbA1c* Glycated haemoglobin, *CBC* Complete blood count

### Affordability of the study medicines and diagnostic tests of interest

#### Selected medicines

The only affordable medicines were parenteral benzathine penicillin 2.4 MU (0.3 days’ wages), oral furosemide 40 mg (0.5 days’ wages), glibenclamide 5 mg (0.7 days’ wages), bendrofluazide 5 mg (0.7 days’ wages), atenolol 50 mg (0.7 days’ wages), cardiac aspirin 75 mg (0.9 days’ wages), digoxin 0.25 mg (1.4 days’ wages), metformin 500 mg (2.8 days’ wages), captopril 25 mg (2.8 days’ wages) and nifedipine 20 mg (2.8 days’ wages). The most unaffordable medicines were enoxaparin 80 mg (53.5 days’ wages), enoxaparin 60 mg (41.2 days’ wages) and UFH (38.5 days’ wages).

Monthly management of an adult diabetic patient with the cheapest oral hypoglycaemic agents (Glibenclamide 5 mg and metformin 500 mg), ACEI (captopril 25 mg), statin (simvastatin 20 mg) and anti platelet drug (cardiac aspirin 75 mg) would cost a total of 15.8 USD; equivalent to 12.2 days’ wages. The monthly cost increased to 19.1 USD or 14.7 days’ wages if glimepiride, a newer generation sulphonylurea was used or to 21.3 USD or 16.4 days’ wages if pre mixed insulin was used instead of a sulphonylurea.

Management of hypertension co-morbidity by adding the cheapest CCB (nifedipine 20 mg) would cost 19.4 USD or 14.9 days’ wages. Secondary prevention of CVD using the cheapest selective beta blocker (bisoprolol 5 mg), ACEI (captopril 25 mg), statin (simvastatin 20 mg) and anti platelet drug (cardiac aspirin 75 mg) would cost a total of 18.3 USD per month which is equivalent to 14.1 days’ wages (summarised in Table 4).

**Table 4:** Median prices and affordability of the DM-CVD lowest priced generic drugs in both private hospitals and pharmacies

Medicine (*N* = 37)	Median (IQR) price/tab in Ug Shs	Median price/tab in USD	IRP in USD	MPR	Monthly cost in USD	Days’ wages^c^
A: Affordable drugs according to calculated days’ wages (≤ 3)						
Benzathine penicillin 2.4 MU	1500 (1500–2000)	0.42	0.3241	1.2	0.4	0.3
Furosemide tablet 40 mg	100 (100–100)	0.02	0.0061	3.3	0.6	0.5
Glibenclamide 5 mg	100 (100–200)	0.03	0.0042	7.1	0.9	0.7
Bendrofluazide 5 mg	100 (100–100)	0.03	0.0072	4.2	0.9	0.7
Atenolol 50 mg	100 (100–200)	0.03	0.0106	2.8	0.9	0.7
Cardiac aspirin 75 mg	150 (150–200)	0.04	0.0196	2.0	1.2	0.9
Digoxin tablet 0.25 mg	200 (200–300)	0.06	0.0148	4.1	1.8	1.4
Metformin 500 mg	200 (200–300)	0.06	0.0168	3.6	3.6	2.8
Captopril 25 mg	200 (200–300)	0.06	0.0216	2.8	3.6	2.8
Nifedipine 20 mg	200 (100–250)	0.06	0.0233	2.6	3.6	2.8
B: Unaffordable drugs according to the calculated days’ wages (> 3)						
Amlodipine 5 mg	500 (400–500)	0.14	0.0321	4.4	4.2	3.2
Warfarin 5 mg	500 (500–500)	0.14	0.0369	3.8	4.2	3.2
Glimepiride 2 mg	500 (400–1000)	0.14	–	–	4.2	3.2
Spironolactone 25 mg	500 (400–600)	0.14	0.0398	3.5	4.2	3.2
Furosemide i.v vial 20 mg	1000 (1000–1500)	0.28	0.0623	4.5	4.2	3.2
Hydralazine tablet 25 mg	300 (225–450)	0.08	0.0376	2.1	4.8	3.7
Telmisartan 40 mg	600 (500–800)	0.17	–	–	5.1	3.9
Bisoprolol 5 mg	600 (500–800)	0.17	–	–	5.1	3.9
Pioglitazone 30 mg	600 (500–800)	0.17	–	–	5.1	3.9
Losartan 50 mg	700 (600–800)	0.19	0.2443	0.8	5.7	4.4
Soluble insulin 1vial	22,000 (20000–25,000)	6.11	0.7723	7.9	6.1	4.7
Amlodipine 10 mg	750 (700–800)	0.21	0.1414	1.5	6.3	4.9
Intermediate insulin 1vial	23,000 (20000–25,000)	6.39	0.7723	8.3	6.4	4.9
Pre mixed insulin 1vial	23,000 (21000–25,000)	6.39	0.6143	10.4	6.4	4.9
Clopidogrel 75 mg	800 (700–1100)	0.22	0.0736	3.0	6.6	5.1
Losartan H 62.5 mg	800 (700–900)	0.22	–	–	6.6	5.1
Telmisartan H 52.5 mg	800 (700–1000)	0.22	–	–	6.6	5.1
Nebivolol 5 mg	1000 (900–1300)	0.28	–	–	8.4	6.5
Simvastatin 20 mg	1000 (900–1500)	0.28	0.0252	11.1	8.4	6.5
Isosorbide mono-nitrate 10 mg	1000 (985–1600)	0.28	0.0705	4.0	8.4	6.5
Atovastatin 20 mg	1200 (1000–2000)	0.33	0.0640	5.2	9.9	7.6
Rosuvastatin 10 mg	1200 (1000–2000)	0.33	–	–	9.9	7.6
Carvedilol 6.25 mg	700 (600–900)	0.19	0.0445	4.3	11.4	8.8
Metformin 1000 mg	700 (500–800)	0.19	–	–	11.4	8.8
UFH^b^ 5000 units	20,000 (18000–20,000)	5.56	0.9055	6.1	50.0	38.5
LMWH^a^ 60 mg	38,500 (34500–45,000)	10.69	2.8751	3.7	53.5	41.2
LMWH^a^ 80 mg	50,000 (45000–60,000)	13.89	5.8025	2.4	69.5	53.5

*IQR* Inter-quartile range, ^a^ *LMWH* Low molecular weight heparin, ^b^ *UFH* Unfractionated heparin

^c^Days’ wages-number of days’ wages to cover the monthly costs of the selected drug

### Median cost, pricing of the available LPG medicines and their comparison with the available originator medicines

With regard to pricing as reflected by the MPR, the only reasonably priced medicines were parenteral benzathine penicillin (1.2), losartan 50 mg (0.8) and amlodipine 10 mg (1.5). The MPR ranged from 0.8 for losartan 50 mg to 11.1 for simvastatin (summarised in Table 4). With the exception of Glucophage® (metformin) 1 g, Insulitard® (intermediate insulin) and Mixtard® (pre mixed insulin), all of the available originator medicine brands cost more than the available LPG medicine brands. One originator brand (Adalat 30 mg) cost up to 10 times the cost of the available LPG brand (nifedipine 20 mg) (summarised in Table 5).

**Table 5:** Comparison between the median prices of the originator and lowest priced generic medicines

Originator medicine (OM)	Median (IQR) price of OM in UgX	Median (IQR) price of OM in USD	Name of LPG medicine	Median (IQR) of the LPG medicine (UgX)	Median (IQR) price of LPG in USD
Micardis 40 mg	3700 (2000–4000)	1.0 (0.6–1.1)	Telmisartan 40 mg	600 (500–800)	0.2 (0.1–0.2)
Co-Micardis 40/12.5 mg	4000 (2000–4500)	1.1 (0.6–1.3)	Telmisartan-H	800 (700–1000)	0.2 (0.2–0.3)
Adalat 30 mg	2000 (1500–2500)	0.6 (0.5–0.7)	Nifedipine 20 mg	200 (100–250)	0.1 (0.02–0.1)
Norvasc 5 mg	1000 (1000–2600)	0.3 (0.3–0.7)	Amlodipine 5 mg	500 (400–500)	0.1 (0.1–0.1)
Norvasc 10 mg	1500 (1250–2800)	0.4 (0.3–0.8)	Amlodipine 10 mg	750 (700–800)	0.2 (0.2–0.2)
Concor 5 mg	2500 (1800–2500)	0.7 (0.5–0.7)	Bisoprolol 5 mg	600 (500–800)	0.2 (0.1–0.2)
Lipitor 20 mg	3000 (2000–4000)	0.8 (0.6–1.1)	Atovastatin 20 mg	1200 (1000–2000)	0.3 (0.3–0.6)
Crestor 10 mg	3000 (2500–3500)	0.8 (0.7–1)	Rosuvastatin 10 mg	1200 (1000–2000)	0.3 (0.3–0.6)
Bayer aspirin 100 mg	400 (300–500)	0.1 (0.1–0.1)	Cardiac aspirin 75 mg	150 (150–200)	0.04 (0.04–0.1)
Glucophage 500 mg	400 (300–500)	0.1 (0.1–0.1)	Metformin 500 mg	200 (200–300)	0.1 (0.1–0.1)
Glucophage 1000 mg	600 (525–800)	0.2 (0.2–0.2)	Metformin 1000 mg	700 (500–800)	0.2 (0.1–0.2)
Amaryl 2 mg	1800 (1000–2000)	0.5 (0.3–0.6)	Glimepiride 2 mg	500 (400–1000)	0.1 (0.1–0.3)
Actrapid 1 vial	23,000 (22000–25,000)	6.4 (6.1–6.9)	Soluble insulin 1 vial	22,000 (20000–25,000)	6.1 (5.6–6.9)
Insulitard 1 vial	23,000 (22000–25,000)	6.4 (6.1–6.9)	Intermediate insulin	23,000 (20000–25,000)	6.4 (5.6–6.9)
Mixtard 1 vial	23,000 (22000–25,000)	6.4 (6.1–6.9)	Pre mixed insulin	23,000 (21000–25,000)	6.4 (5.8–6.9)
Clexane 60 mg	42,000 (35000–46,800)	11.7 (9.7–13)	LMWH 60 mg	38,500 (34500–45,000)	10.7 (9.6–12.5)
Clexane 80 mg	60,000 (55000–69,000)	16.7 (15.3–19.2)	LMWH 80 mg	50,000 (45000–60,000)	13.9 (12.5–16.7)

*OM* Originator medicine, *LPG* Lowest priced generic, *IQR* Inter-quartile range, *LMWH* Low molecular weight heparin, *UgX* Uganda shillings, *USD* US dollars

#### Selected diagnostic tests

The only affordable tests were random blood glucose measurement (1.1 days’ wages), urinalysis (1.3 days’ wages), serum ketone measurement (2.1 days’ wages), serum uric acid measurement (2.1 days’ wages), serum creatinine measurement (2.4 days’ wages) and serum urea measurement (2.4 days’ wages). The most unaffordable diagnostic tests were echocardiography and serum natriuretic peptides that cost 33.1 and 40.6 days’ wages respectively.

The cost of performing the WHO recommended tests for CV risk assessment and management (proteinuria, ECG, FBG, lipid profile and serum electrolytes measurement) was 33.5 USD or 25.8 days’ wages if urinalysis was used. The cost increased to 44.3 USD or 34.1 days’ wages if microalbuminuria was used instead of urinalysis. The WHO CV risk monitoring tests (lipid profile, FBG and proteinuria) cost 23.6 USD/18.2 days’ wages or 12.8 USD/7.1 days’ wages when using microalbuminuria or urinalysis respectively. Annual monitoring of adult diabetic patients using HbA1c measurement at least twice a year, annual ECG, microalbuminuria and lipid profile assessment as recommended by the ADA guidelines of diabetes management would cost 58.2 USD or 44.8 days’ wages (summarised in Table 6).

**Table 6:** Cost and affordability of DM-CVD selected diagnostic/screening tests in the private hospitals

Test	Median (IQR) price in UgX	Median price in USD	Monthly cost in USD	Days’ wages for test
A: Affordable diagnostic tests				
RBG	5000 (5000–6950)	1.4	1.4	1.1
Urinalysis	6250 (5000–10,000)	1.7	1.7	1.3
Serum ketones	10,000 (10000–10,000)	2.8	2.8	2.1
Serum uric acid	10,000 (10000–15,500)	2.8	2.8	2.1
Serum urea	11,000 (10000–19,900)	3.1	3.1	2.4
Serum creatinine	11,000 (10000–20,000)	3.1	3.1	2.4
B: Unaffordable diagnostic tests				
CBC	15,000 (13000–20,000)	4.2	4.2	3.2
Serum electrolytes	25,000 (15000–30,000)	6.9	6.9	5.3
Coagulation profile	30,000 (20000–30,000)	8.3	8.3	6.4
Chest X-ray	30,000 (25000–45,000)	8.3	8.3	6.4
Lipid profile	35,000 (30000–41,000)	9.7	9.7	7.5
Liver function tests	36,000 (27500–47,850)	10.0	10.0	7.7
Serum HbA1c	40,000 (35000–40,000)	11.1	11.1	8.6
Microalbuminuria	45,000 (45000–45,000)	12.5	12.5	9.6
Electrocardiography	50,000 (40000–52,000)	13.8	13.8	10.7
Serum troponin	53,000 (45000–60,000)	14.7	14.7	11.3
Thyroid function tests	100,000(77500–149,000)	27.8	27.8	21.4
Echocardiography	155,000 (100000–175,000)	43.1	43.1	33.1
Serum natriuretic peptides	190,000 (150000–275,000)	52.8	52.8	40.6

*RBG* Random blood glucose, *CBC* Complete blood count, *HbA1c* Glycated haemoglobin, *UgX* Uganda shillings, *USD* US dollars

## Discussion

The ACCODAD study sought to provide contemporary data about the availability, cost and affordability of medicines and diagnostic tests integral in the management of DM and CVD in Uganda, a low income developing country in East Africa. To the best of our knowledge, this is the largest study in Uganda to comprehensively investigate the availability, cost and affordability of a substantial number of medicines and diagnostic tests that play a fundamental role in optimal DM and CVD management in clinical practice.

### Availability of medicines and diagnostic tests

In our study, low and moderate availability (availability of <80% in all study sites) was reported in 38.1% of the medicines and 57.9% of the diagnostic tests of interest. Several similar studies assessing access to medicines and diagnostic tests of NCDs in LMIC have reported similar findings of low availability of medicines and diagnostic tests especially in the public sector [5–10].

In the study reported from Western Cameroon, high availability defined as availability ≥80% was only noted with 6 (27%) of the surveyed medicines (parenteral benzathine penicillin 2.4 MU, oral furosemide 40 mg, glibenclamide 5 mg, Actrapid/soluble insulin, metformin 500 mg and Mixtard). The majority of rural study sites had low availability of medicines [6]. In comparison with our study, with the exception of the soluble and pre mixed insulin, high availability of >80% was documented with parenteral benzathine penicillin, oral furosemide, glibenclamide and metformin.

In another study that assessed the availability, pricing and affordability of 5 key cardiovascular medicines (atenolol, captopril, hydrochlorothiazide, losartan and nifedipine) in 36 LMIC (Uganda inclusive), upper middle income and high income countries found an overall poor availability of these medicines. Only 26.3 and 57.3% of the medicines were available in the public and private sector respectively [7]. In another similar multicentre study involving 90 primary care facilities in 8 LMIC, some of the 12 surveyed CVD and DM medicines were not available in some countries. Soluble and long acting insulin was absent in all study sites in Benin, Eriteria, Bhutan and Vietnam. Isosorbide mono nitrate was absent in Benin, Eriteria, Sudan and Bhutan and simvastatin and amlodipine were absent in Eriteria and Bhutan [10]. Low availability of nitrates, intermediate and pre-mixed insulin was also reported by our study (27.8, 34.7 and 60.1% respectively).

With regard to the availability of diagnostic tests, cross sectional studies in LMIC had reported similar findings of low availability [5–9]. In one of these studies performed in the Western Cameroon in 2012, high availability defined as availability ≥80% was only noted with 50% of the surveyed diagnostic tests (RBG, urinalysis, serum creatinine, serum urea and CBC). Serum electrolytes, lipid profile and uric acid tests, HbA1c tests and ECG were only available in 60, 40, 20 and 10% of all the study sites [6].

Another multicentre study performed in 90 primary care centres of 8 LMIC (Benin, Bhutan, Eritrea, Sri Lanka, Sudan, Suriname, Syria, and Vietnam), lipid profile testing was available only in 33, 25, 20, 14 and 8% of all study sites in Sudan, Benin, Suriname, Syria and Sri Lanka. Low availability of serum creatinine tests was also reported in the majority of the study sites. Of all study sites, serum creatinine tests were available in 58% in Sudan, 33% in Benin, 10% in Suriname and 8% in Sri Lanka. Lipid profile tests were absent in Eriteria and Vietnam while serum creatinine tests were absent in all study sites in Eriteria, Bhutan and Syria. Serum troponin tests were absent in all the countries except Benin and Sudan where it was available in only 8% of the study sites [10].

Comparing with our study, we also reported similar findings of low availability of uric acid, HbA1c, ECG and troponin tests (all <60%).

### Affordability of medicines and diagnostic tests of interest

The majority of surveyed medicines and diagnostic tests have also been reported to be unaffordable in most studies in LMIC, similar to the findings of our study. Our study findings documented that only 27% of the surveyed medicines and 32% of the surveyed diagnostic tests were affordable in Uganda.

In comparison, only 7 (32%) of the studied medicines (oral aspirin 500 mg, hydrochlorothiazide 50 mg, furosemide 40 mg, nifedipine 10 and 20 mg, glibenclamide 5 mg and metformin 500 mg) were affordable according to the study definition of affordability i.e. monthly cost of a medicine of ≤1 days’ wages of a lowest paid public servant in the study performed in Western Cameroon [6]. The most unaffordable medicines in this study were Mixtard, simvastatin 20 mg and heparin 5000 IU costing 18.7 days’ wages, 30.5 days’ wages and 182.36 days’ wages respectively [6]. In our study, only 6 (16%) medicines cost less than a days’ wages (parenteral benzathine penicillin, oral furosemide, glibenclamide, bendrofluazide, atenolol, and cardiac aspirin).

In another multicentre study by Mendis S et al. in 6 LMIC (Bangladesh, Malawi, Sri Lanka, Brazil, Nepal and Pakistan) assessing availability and affordability of 32 medicines essential in CVD, DM, asthma, glaucoma and palliative care, anti hypertensive management using hydrochlorothiazide monotherapy and glycaemic therapy using either glibenclamide or metformin was affordable in all the 6 countries (cost ≤1 days’ wages). Using intermediate insulin for DM management was unaffordable with a monthly cost equivalent to 2.8 days’ wages in Brazil, 4.7 days’ wages in Pakistan, 6.1 days’ wages in Sri Lanka, 7.3 days’ wages in Nepal and 19.6 days’ wages in Malawi. Secondary prevention of CVD with an oral aspirin, statin, beta blocker and ACEI in this study was also unaffordable in some countries. This combination using a generic medicine would cost ≤1.6 days’ wages in Sri Lanka and Bangladesh, ≤ 6.1 days’ wages in Brazil, Pakistan and Nepal and 18.4 days’ wages in Malawi. The cost of treatment in Malawi would increase to 48.8 days’ wages if an innovator statin brand was used [5]. In our study, the monthly cost of combination therapy used in secondary CVD prevention using the cheapest beta blocker, ACEI, statin and cardiac aspirin was 14.1 days’ wages; almost similar to the cost in Malawi.

Regarding diagnostic tests, only urinalysis and random blood glucose tests cost less than 1.5 days’ wages in the study by Jingi A et al. in Western Cameroon. Other key tests recommended by the WHO in optimal DM and CVD workup in this study like lipid profile tests cost 3.1–3.6 days’ wages and ECG cost 10.7 days’ wages. Glycated haemoglobin (HbA1c), an important test in diagnosis and monitoring of glycaemic control in diabetes care cost 12.6 days’ wages [6]. Comparing to our study findings, serum electrolytes, lipid profile test, HbA1c test and ECG cost 5.3 days’ wages, 7.5 days’ wages, 8.6 days’ wages and 10.7 days’ wages respectively.

There are several plausible explanations for the low availability of medicines and diagnostic tests especially in the public hospitals in our study. Some of these key medicines are not included in the 2012 national essential medicine list and the 2016 Uganda Clinical guidelines of management of NCDs. Inadequate allocation of funds to the health sector to enable procurement of most essential medicines and diagnostic tests, poor stock maintenance, forecast inaccuracy and inefficient distribution systems from the national central procurement institution could also explain the low availability in the public and private sectors.

The study finding of the majority of medicines and diagnostic tests being unaffordable in the private sector could be explained by the lack of local legislation to regulate the maximum retail prices of medicines and a vibrant local pharmaceutical industry sector to produce cheap quality generic medicines for chronic diseases.

## Conclusions

The ACCODAD study evidently demonstrates that the majority of medicines and diagnostic tests important in DM and CVD care are largely unavailable and unaffordable in Uganda. These study findings offer contemporary information to guide pragmatic approaches to address the inequity in access to affordable medicines and diagnostic tests that are essential in DM and CVD care in Uganda. More emphasis should be directed towards improving access to these essential medicines and diagnostic tests in the public sector by ensuring their procurement by the mandated central procurement institution (NMS): ARBs, statins, warfarin, heparins, nitrates, lipid profile, HbA1c, uric acid, troponin, coagulation profile and thyroid function tests.

Concerted efforts to reduce the cost of UFH, LMWH, new generation beta blockers, statins and insulin in the private sector should be encouraged. Generally, availability and affordability of these medicines and diagnostic tests can be improved in Uganda by improving procurement efficiency, stock handling, forecast accuracy, ensuring equitable financing to the health sector and regular updating of the national essential drug list and management guidelines. Strategies like boosting local pharmaceutical production of high quality generic DM and CVD medicines and introduction of local laws to regulate retail prices of medicines can assist in reducing the cost of these medicines. Due to the high costs of secondary and tertiary DM and CVD management, national policies to promote primary prevention strategies of NCD should be widely adopted.

### Study limitations

Being a point in time study, variations in availability, pricing and affordability of the medicines and diagnostic tests was not put into consideration. We were unable to obtain the procurement prices from the central procurement institution, NMS to obtain the prices of the medicines and diagnostic tests in the public hospitals. Using the daily wage of the lowest paid unskilled government to calculate affordability of medicines and diagnostic tests has its limitations because a significant proportion of the Ugandan population earns less than this amount. Despite these limitations, the study has its strengths. The standardised WHO/HAI methodology that was used has been widely validated.

## Abbreviations

ACCODAD: Access to Cardiovascular diseases, Chronic Obstructive pulmonary disease, Diabetes mellitus and asthma drugs and diagnostics
ACEI: Angiotensin converting enzyme inhibitors
ADA: American Diabetes Association
ARB: Angiotensin II receptor blockers
CCB: Calcium channel blockers
CD: Communicable diseases
CVD: Cardiovascular diseases
DM: Diabetes mellitus
ECG: Electrocardiography
ECHO: Echocardiography
ESC: European Society of Cardiology
FBG: Fasting blood glucose
HAI: Health Action International
HbA1c: Glycated haemoglobin
HT: Hypertension
IDF: International diabetes federation
IRP: International reference price
LMIC: Low and middle income countries
LPG: Lowest priced generic
MPR: Median price ratio
NCD: Non communicable diseases
NDA: National Drug Authority
NMS: National Medical Stores
OHA: Oral hypoglycaemic agents
UFH: Unfractionated heparin
UgX: Uganda shillings
USD: US dollars
WHO: World Health Organisation
